# Circulating Neurovascular Guidance Molecules and Their Relationship with Peripheral Microvascular Impairment in Systemic Sclerosis

**DOI:** 10.3390/life12071056

**Published:** 2022-07-14

**Authors:** Eloisa Romano, Irene Rosa, Bianca Saveria Fioretto, Marco Matucci-Cerinic, Mirko Manetti

**Affiliations:** 1 Division of Rheumatology, Department of Experimental and Clinical Medicine, University of Florence, 50134 Florence, Italy; eloisa.romano@unifi.it (E.R.); biancasaveria.fioretto@unifi.it (B.S.F.); marco.matuccicerinic@unifi.it (M.M.-C.); 2 Section of Anatomy and Histology, Department of Experimental and Clinical Medicine, University of Florence, 50134 Florence, Italy; irene.rosa@unifi.it; 3 Unit of Immunology, Rheumatology, Allergy and Rare Diseases (UnIRAR), IRCCS San Raffaele Hospital, 20132 Milan, Italy

**Keywords:** systemic sclerosis, scleroderma, neurovascular guidance molecules, sNRP1, Sema3E, Slit2, peripheral microvasculopathy, nailfold videocapillaroscopy, ischemic digital ulcers

## Abstract

Systemic sclerosis (SSc, scleroderma) is a complex connective tissue disease whose earliest clinical manifestations are microvascular tone dysregulation and peripheral microcirculatory abnormalities. Following previous evidence of an association between circulating neurovascular guidance molecules and SSc disturbed angiogenesis, here, we measured the levels of soluble neuropilin 1 (sNRP1), semaphorin 3E (Sema3E), and Slit2 by enzyme-linked immunosorbent assay in serum samples from a large case series of 166 SSc patients vs. 110 healthy controls. We focused on their possible correlation with vascular disease clinical features and applied logistic regression analysis to determine which of them could better reflect disease activity and severity. Our results demonstrate that, in SSc: (i) sNRP1 is significantly decreased, with lower sNRP1 serum levels correlating with the severity of nailfold videocapillaroscopy (NVC) abnormalities and the presence of ischemic digital ulcers (DUs); (ii) both Sema3E and Slit2 are increased, with Sema3E better reflecting early NVC abnormalities; and (iii) higher Sema3E correlates with the absence of DUs, while augmented Slit2 associates with the presence of DUs. Receiver operator characteristics curve analysis revealed that both circulating sNRP1 and Sema3E show a moderate diagnostic accuracy. Moreover, logistic regression analysis allowed to identify sNRP1 and Sema3E as more suitable independent biomarkers reflecting the activity and severity of SSc-related peripheral microvasculopathy.

## Introduction

Systemic sclerosis (SSc, scleroderma) is a severe multisystem connective tissue disease that is dominated by the pathogenic triad of autoimmunity, widespread peripheral microvasculopathy, and progressive cutaneous and visceral fibrosis eventually leading to substantial organ dysfunction [1,2,3]. Peripheral microvascular tone dysregulation, manifesting with Raynaud’s phenomenon (RP), as well as microcirculatory abnormalities, mirrored by nailfold capillaroscopic changes, represent the earliest clinical manifestations of SSc and may precede both cutaneous and organ fibrosis by months or years [1,2,3]. Such a microvascular dysfunction is characterized by vascular repair impairment and defective angiogenesis culminating in capillary network disruption, and frequently leads to significant peripheral ischemic manifestations such as digital ulcers (DUs) [4,5]. SSc-related ischemic DUs are disabling and painful lesions that are often refractory to treatment and may result in severe complications including infections and gangrene, thus heavily compromising patients’ quality of life [4,5].

It is broadly recognized that blood vessels and nerves share several anatomical and structural similarities, both being organized in complex and branched networks following parallel routes and requiring precise control over their guidance and growth [6,7]. Interestingly, it has been shown that, during their development, the nervous and vascular systems communicate with each other in a neurovascular crosstalk, with vessels producing signals that are able to attract axons to track alongside the pioneer vessels and, in turn, nerves releasing signals able to guide blood vessel growth [6,7,8,9,10]. In this context, several molecules with attractive and repulsive properties, here referred to as neurovascular guidance molecules, have been reported to regulate the sprouting of both nerves and blood vessels [8,9,10], and to be involved in different pathologic processes including tumor growth/metastasis, nephropathy, as well as autoimmune diseases such as rheumatoid arthritis and systemic lupus erythematosus [11,12,13,14,15,16,17]. As far as SSc is concerned, members of semaphorin/plexin/neuropilin and slit/roundabout families have been recently implicated in SSc-related disturbed neuroendothelial control of vascular tone, peripheral microvasculopathy, and defective angiogenesis, with their circulating levels being significantly associated with different vascular disease manifestations [9,18,19,20,21,22]. In particular, previous studies from our group demonstrated that circulating levels of the angiogenic regulator soluble neuropilin 1 (sNRP1) progressively decreased in SSc patients, reaching the lowest values in those with the most severe architectural microvascular changes, while serum levels of semaphorin 3E (Sema3E) and Slit2 were increased, especially in patients with early peripheral vascular involvement [18,20,21,22]. Although variations in the levels of these neurovascular guidance molecules have been proposed as a useful tool for evaluating microcirculatory abnormalities at different stages of SSc [9,18,20,21,22], it is worth noting that the aforementioned studies have been conducted to assess a single molecule at a time and in relatively small groups of patients. Based on this scientific background, the objective of the present research work was to measure in parallel circulating levels of sNRP1, Sema3E, and Slit2 by enzyme-linked immunosorbent assay (ELISA) in a larger case series of SSc patients, focusing on the peripheral vascular disease features. Moreover, we carried out a logistic regression analysis to define which of these neurovascular guidance molecules could better and independently reflect the severity of SSc-related peripheral microvasculopathy.

## 2. Materials and Methods

### 2.1. Patients, Controls and Serum Sample Collection

Serum samples were collected from 166 patients fulfilling the American College of Rheumatology (ACR)/European League Against Rheumatism (EULAR) 2013 classification criteria for SSc [23] (144 women and 22 men; mean ± SD age 58.6 ± 13.7 years) and recruited from the Division of Rheumatology and Scleroderma Unit, Azienda Ospedaliero-Universitaria Careggi (AOUC), Florence, Italy. Patients showing symptoms that were common to other autoimmune, rheumatic, and/or connective tissue diseases were excluded from the study. SSc patients were not taking immunosuppressants or other disease-modifying medications at the time of blood sample collection. A total of 110 age- and sex-matched healthy individuals (96 women, 14 men; mean ± SD age 59.2 ± 13.1 years) were used as controls; the presence of primary RP was considered as an exclusion criterion. Fresh venous blood samples from both patients and controls were drawn and left to clot for 30 min before centrifugation at 1500× *g* for 15 min. The serum was then collected and stored in aliquots at −80 °C until use. The study was performed in agreement with the Declaration of Helsinki and approved by the local institutional review board at the AOUC, Florence, Italy (approval number: AOUC 69/13; approval date: 17 June 2013). All the enrolled subjects provided written informed consent.

### 2.2. Clinical Assessment

The patients were classified as limited cutaneous SSc (*n* = 111) or diffuse cutaneous SSc (*n* = 55) according to the criteria of LeRoy et al. [24], and phenotypically assessed as recommended [25]. All the patients reported the occurrence of RP. At the time of blood sampling, the presence of ischemic DUs on the fingertips and other finger areas was documented, and microvascular abnormalities on all 10 fingers were assessed by nailfold videocapillaroscopy (NVC). Specifically, after being allowed to adapt to room temperature (20–22 °C) for a minimum of 15 min, the patients were subjected to the analysis of their nailfolds in order to evaluate the presence of pericapillary edema, microhemorrhages, enlarged and giant capillaries, ramified or bushy capillaries, disorganization of the vascular distribution, and loss of capillaries. The 3 different NVC patterns were identified as follows: (i) early NVC pattern, with few enlarged/giant capillaries and capillary microhemorrhages, no evident capillary loss, and a relatively well-preserved capillary bed; (ii) “active” NVC pattern, featuring giant capillaries and capillary microhemorrhages, absence/presence of few ramified capillaries, moderate capillary loss, and mild disorganization of the capillary structure; and (iii) “late” NVC pattern, with irregular capillary enlargement, absence/presence of few giant capillaries, no microhemorrhages, frequent ramified/bushy capillaries, severe capillary loss with large avascular areas, and disorganization of the normal capillary architecture [26]. The main demographic, clinical, and serological characteristics of SSc patients are summarized in Table 1.

### 2.3. Assay for Serum sNRP1

The serum levels of sNRP1 were assessed by commercial quantitative colorimetric sandwich ELISA (catalog No. E2101Hu; BT Lab Bioassay Technology Laboratory, Birmingham, UK), according to the manufacturer’s protocol. Each sample was measured in duplicate. Briefly, 50 µL of each standard (already containing the anti-NRP1 biotinylated antibody) and 40 µL of each sample were added to the 96-well microtiter plate that was precoated with a human antibody that was specific to NRP1. Next, 10 µL of biotinylated human anti-NRP1 antibody was added to sample wells, while 50 µL of streptavidin-horseradish peroxidase (HRP) was added to both standard and sample wells. After 60 min at 37 °C, the plate was washed for 5 times, and each well was incubated for 10 min at 37 °C in the dark with 50 µL of substrate solution A plus 50 µL of substrate solution B. The enzyme-substrate reaction was stopped with 50 µL/well of stop solution and the optical densities (OD) were determined within 10 min using a microplate reader that was set to 450 nm. The detection range and the sensitivity of the assay were 0.1–40 ng/mL and 0.054 ng/mL, respectively.

### 2.4. Assay for Serum Sema3E 

The Sema3E serum levels were measured by a commercial colorimetric sandwich ELISA (catalog No. LS-F7925; LifeSpan Biosciences, Seattle, WA, USA), according to the manufacturer’s instructions. Once both standards and samples (100 µL/well) were added to the 96-well microtiter plate that was precoated with a capture antibody specific for Sema3E, the plate was left to incubate for 1 h at 37 °C. The liquid was then aspirated without washing and the wells were incubated for 1 h at 37 °C with 100 µL of detection reagent A working solution (biotin-conjugated detection antibody). The plate was subsequently washed 3 times and incubated for 30 min at 37 °C with 100 µL/well of detection reagent B working solution (HRP conjugate). Following an additional 5 washes, the reaction was firstly developed in the dark with 90 µL/well of tetramethylbenzidine (TMB) substrate solution for 10–20 min at 37 °C, and finally terminated by applying 50 µL of the sulfuric acid stop solution. The absorbance of each well was read using a microplate reader at 450 nm. The serum levels of Sema3E were read from a standard curve that was prepared using a lyophilized protein standard that was reconstituted with the standard diluent included in the kit. The detection range of the assay was 0.312–20 ng/mL, while its sensitivity was less than 0.114 ng/mL. The serum Sema3E concentration was determined by comparing the OD of each sample to the standard curve. Each sample was measured in duplicate.

### 2.5. Assay for Serum Slit2

The Slit2 levels in serum samples were quantified using a commercial colorimetric sandwich ELISA (catalog No. MBS703756; MyBiosource, San Diego, CA, USA) according to the manufacturer’s protocol. Each sample was measured in duplicate. Briefly, the standards and samples (100 µL/well) were added to the 96-well microtiter plate that was precoated with a specific anti-Slit2 capture antibody, incubated for 2 h at 37 °C, and subsequently removed without washing. Next, the plate was incubated for 1 h at 37 °C with 100 µL/well of the biotin-conjugated antibody, washed 3 times, and then left for 1 h at 37 °C with 100 µL of the avidin-conjugated HRP solution. Following 5 additional washes, the enzyme-substrate reaction was developed in the dark by incubating the plate with 90 µL/well of TMB for 15–30 min at 37 °C, and then terminated by applying 50 µL/well of stop solution. The color change was measured spectrophotometrically at a wavelength of 450 nm within 5 min. The Slit2 concentration in the samples was then determined by comparing the OD of the samples to those of the standard curve. The detection range of the assay was 0.78–50 ng/mL, while the sensitivity was less than 0.195 ng/mL.

### 2.6. Statistical Analysis

Statistical analysis was conducted with the SPSS software for Windows Version 28.0 (SPSS, Chicago, IL, USA). Descriptive statistics for continuous variables were reported as the mean ± SD or median and interquartile range (IQR), while descriptive statistics for categorical variables were expressed as number and percentage. To verify the accuracy of serum sNRP1, Sema3E, and Slit2 levels for the diagnosis of SSc disease, the test performance in terms of sensitivity (ability of the test to identify true positive subjects) and specificity (ability of the test to identify true negative subjects) was evaluated for each molecule by performing receiver operator characteristics (ROC) curve analysis followed by the estimation of the area under the curve (AUC). According to Swets classification, if the AUC = 0.5 the test is not accurate, it is poorly accurate for 0.5 < AUC ≤ 0.7, moderately accurate for 0.7 < AUC ≤ 0.9, highly accurate for 0.9 < AUC < 1, and perfect when AUC = 1 [27]. Youden’s index (=Sensitivity − [1 − Specificity]) was also applied to maximize both sensitivity and specificity and evaluate the best cutoff value in our experimental data distributions. The non-parametric Mann–Whitney U test for independent samples was used to analyze sNRP1, Sema3E, or Slit2 serum differences between two groups. Since Mann–Whitney U test analyses revealed that circulating levels of sNRP1, Sema3E, and Slit2 were significantly different in SSc patients according to the NVC pattern and the occurrence of DUs, we performed multiple logistic regression analysis including these three molecules as independent variables and a single dependent variable each time (i.e., NVC pattern and DUs). Odds ratios (ORs) with 95% confidence intervals (95% CIs) were determined. All *p*-values are two-tailed, and *p*-values < 0.05 were considered statistically significant. 

## 3. Results

### 3.1. Serum sNRP1, Sema3E, and Slit2 Levels in SSc Patients

**Figure 1:**
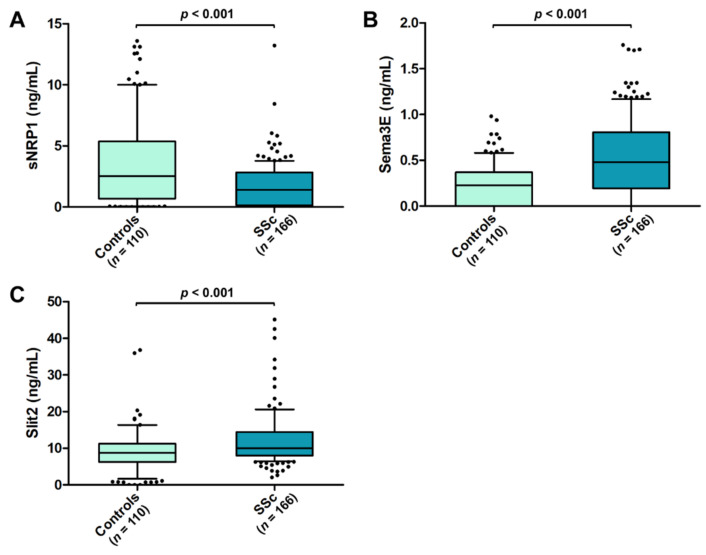
Serum levels of (**A**) sNRP1, (**B**) Sema3E, and (**C**) Slit2 in healthy controls and SSc patients. The data are presented as box plots. Each box denotes the 25th to 75th percentiles. Lines outside the boxes are the 10th and 90th percentiles. Lines inside the boxes denote the median, while dots the outliers; *p*-values are indicated. Sema3E, semaphorin 3E; sNRP1, soluble neuropilin 1; SSc, systemic sclerosis.

Circulating levels of sNRP1 were significantly decreased in SSc patients (median 1.39 ng/mL, IQR 0.14–2.82 ng/mL) compared to the healthy controls (median 2.52 ng/mL, IQR 0.68–5.36 ng/mL; *p* < 0.001; Figure 1A), while both serum Sema3E and Slit2 were found to be significantly increased in SSc with respect to the controls (median 0.48 ng/mL, IQR 0.19–0.80 ng/mL vs. median 0.23 ng/mL, IQR 0.00–0.37 ng/mL for Sema3E; median 9.97 ng/mL, IQR 7.97–14.44 ng/mL vs. median 8.75 ng/mL, IQR 6.22–11.25 ng/mL for Slit2; *p* < 0.001 for both molecules; Figure 1B,C). 

When evaluating the possible association of sNRP1, Sema3E, and Slit2 with different SSc clinical phenotypes, none of the three circulating molecules was found to be significantly different according both to the SSc cutaneous subset (i.e., limited cutaneous SSc and diffuse cutaneous SSc) and to the distinct autoantibody pattern (data not shown).

### 3.2. Diagnostic Accuracy of Circulating sNRP1, Sema3E, and Slit2 for SSc

**Figure 2:**
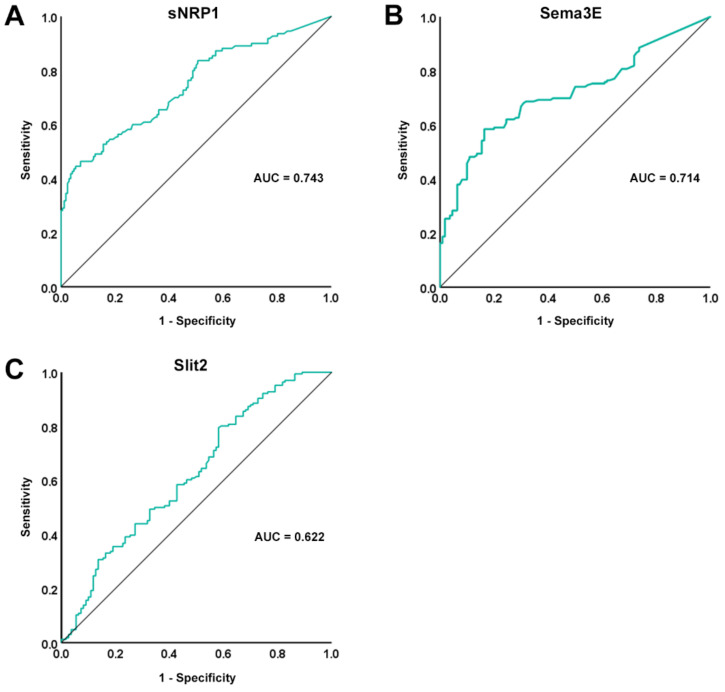
ROC curve (green line) plots for (**A**) sNRP1, (**B**) Sema3E, and (**C**) Slit2 in SSc patients vs. healthy controls. AUC values and reference lines for each curve are shown. AUC, area under the curve; ROC, receiver operator characteristic; Sema3E, semaphorin 3E; sNRP1, soluble neuropilin 1.

The ROC curves and the corresponding AUC were plotted in order to assess the diagnostic accuracy of the three assessed circulating molecules. In particular, the diagnostic accuracy of both sNRP1 and Sema3E was found to be moderate (AUC = 0.743, 95% CI 0.682–0.804 for sNRP1; AUC = 0.714, 95% CI 0.654–0.774 for Sema3E; Figure 2A,B), while Slit2 diagnostic accuracy was proven to be poor (AUC = 0.622, 95% CI 0.554–0.691; Figure 2C). In addition, for sNRP1, the ROC curve analysis revealed a cutoff value of 3.46 ng/mL, with 46.4% sensitivity and 92.8% specificity in discriminating between SSc patients and healthy controls, while the cutoff value for Sema3E was 0.43 ng/mL, with 58.4% sensitivity and 83.6% specificity.

### 3.3. Association of Serum sNRP1, Sema3E, and Slit2 Levels with the Severity of SSc-Related Peripheral Microvascular Damage

**Figure 3:**
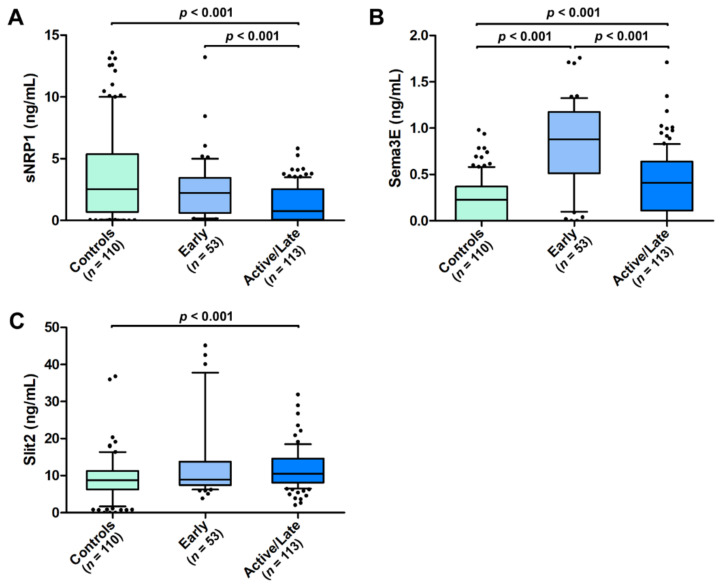
Serum levels of (**A**) sNRP1, (**B**) Sema3E, and (**C**) Slit2 in healthy controls and SSc patients stratified according to early and active/late nailfold videocapillaroscopic patterns. The data are presented as box plots. Each box denotes the 25th to 75th percentiles. Lines outside the boxes are the 10th and 90th percentiles. Lines inside the boxes denote the median, while dots the outliers; *p* values are indicated. Sema3E, semaphorin 3E; sNRP1, soluble neuropilin 1.

As a measure of the severity of peripheral microvascular damage, we further explored the presence of a possible association of circulating sNRP1, Sema3E, and Slit2 with both the NVC pattern and the occurrence of ischemic DUs. As far as NVC is concerned, circulating sNRP1 levels were found to be lower in SSc patients with an active/late NVC pattern (median 0.76 ng/mL, IQR 0.07–2.53 ng/mL) when compared with those with an early NVC pattern (median 2.23 ng/mL, IQR 0.60–3.44 ng/mL; *p* < 0.001; Figure 3A). A similar difference was found for Sema3E (median 0.41 ng/mL, IQR 0.11–0.64 ng/mL for active/late NVC vs. median 0.88 ng/mL, IQR 0.51–1.15 ng/mL for early NVC; *p* < 0.001; Figure 3B), while no difference was detected for Slit2 according to the NVC pattern (median 10.51 ng/mL, IQR 8.08–14.59 ng/mL for active/late NVC vs. median 8.92 ng/mL, IQR 7.45–13.75 ng/mL for early NVC; Figure 3C). When compared to healthy controls, significantly different serum levels of all the three molecules were found in SSc patients with the active/late NVC pattern, with a decrease in sNRP1 and an increase in both Sema3E and Slit2 (*p* < 0.001 for all comparisons; Figure 3A–C). Conversely, only Sema3E values were significantly different in SSc patients with the early NVC pattern compared to the controls (*p* < 0.001; Figure 3B).

**Figure 4:**
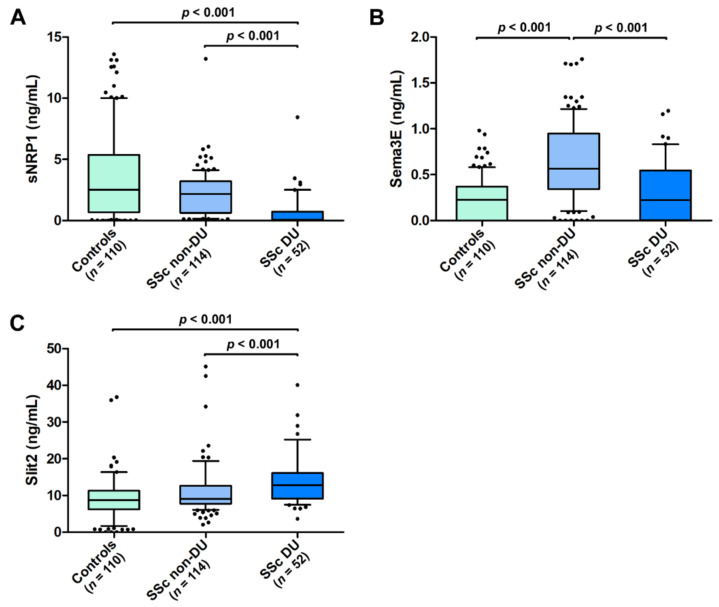
Serum levels of (**A**) sNRP1, (**B**) Sema3E, and (**C**) Slit2 in healthy controls and SSc patients with and without ischemic DUs. The data are presented as box plots. Each box denotes the 25th to 75th percentiles. Lines outside the boxes are the 10th and 90th percentiles. Lines inside the boxes denote the median, while dots the outliers; *p* values are indicated. DUs, digital ulcers; Sema3E, semaphorin 3E; sNRP1, soluble neuropilin 1.

As far as ischemic DUs are concerned, both sNRP1 and Sema3E were significantly lower in SSc patients with DUs (median 0.08 ng/mL, IQR 0.00–0.73 ng/mL for sNRP1 and median 0.22 ng/mL, IQR 0.00–0.54 ng/mL for Sema3E) with respect to those without DUs (median 2.18 ng/mL, IQR 0.62–3.21 ng/mL for sNRP1 and median 0.56 ng/mL, IQR 0.34–0.95 ng/mL for Sema3E; *p* < 0.001 for both molecules; Figure 4A,B). The Slit2 values were, instead, increased in SSc patients with DUs (median 12.75 ng/mL, IQR 9.13–16.11 ng/mL) when compared to those without DUs (median 9.06 ng/mL, IQR 7.79–12.67 ng/mL; *p* < 0.001; Figure 4C). In addition, patients with DUs had lower levels of circulating sNRP1 and higher levels of Slit2 compared to healthy controls (*p* < 0.001 for both comparisons; Figure 4A,C). On the contrary, the Sema3E values were significantly different only in SSc patients without DUs compared to the controls (*p* < 0.001; Figure 4B).

### 3.4. Logistic Regression Model Combining Serum sNRP1, Sema3E, and Slit2 Levels in SSc Patients

Since, when comparing SSc subgroup medians, we found significant differences in circulating sNRP1, Sema3E, and Slit2 according to the severity of the NVC pattern and the presence of ischemic DUs, we finally performed multiple logistic regression analysis combining the serum levels of the three molecules as independent variables, and one of the two abovementioned disease phenotypes as a single dependent variable each time. The results of the logistic regression analysis are shown in Table 2.

## 4. Discussion

Following the previously reported associations between the serum levels of neurovascular guidance molecules and SSc-related peripheral vascular disease manifestations [9,18,20,21,22], the results of the present case-control study, carried out on a large cohort consisting of 166 SSc patients vs. 110 healthy controls, demonstrated that: (i) circulating levels of sNRP1 are significantly decreased in SSc, with lower sNRP1 correlating with the severity of NVC abnormalities and the presence of ischemic DUs; (ii) both circulating Sema3E and Slit2 are increased in SSc, with Sema3E better reflecting early NVC abnormalities; and (iii) higher Sema3E correlates with the absence of DUs, while augmented Slit2 significantly associates with the presence of DUs. Of note, ROC curve analysis revealed that both sNRP1 and Sema3E serum levels show a moderate diagnostic accuracy. In addition, the findings of our logistic regression analysis allowed us to discriminate that, among the three neurovascular guidance molecules, sNRP1 and Sema3E may be better suitable as independent biomarkers reflecting the activity and severity of SSc-related peripheral microvasculopathy. Moreover, the evidence that sNRP1 levels in SSc patients with early NVC pattern are comparable to those in healthy controls but significantly higher than in patients with an active/late NVC pattern may indicate that, rather than being useful for early diagnosis, they might be employed as marker of microvascular disease progression. Conversely, since the Sema3E levels rise, particularly in patients with an early NVC pattern followed by their decrease with progression of microvascular abnormalities, it can be speculated that this molecule may better suited as early diagnostic marker. 

A considerable body of evidence indicates that a dysregulation in neuroendothelial control mechanisms, together with structural and functional peripheral microvascular alterations clinically manifesting with RP and distinctive NVC patterns, represent the initial manifestations of SSc and may precede skin and visceral fibrosis by months or years [9,28,29,30]. Indeed, peripheral microvasculopathy can be easily observed with NVC, a non-invasive technique that is included in the 2013 ACR/EULAR recommendations for the diagnosis and management of SSc that allows both the qualitative and quantitative evaluation of the microcirculation, thus enabling early detection of abnormalities [23,31,32]. Notably, plenty of literature indicates that NVC changes in SSc patients are often accompanied by abnormal levels of angiogenic/angiostatic factors acting on endothelial cells, some of which have been proposed as vascular biomarkers [4,33,34]. In recent years, increasing studies have demonstrated that endothelial cells express neuropilins, plexins, and roundabouts, all axon guidance molecule receptors that interact with their soluble neuroendothelial ligands, such as semaphorins and slits, to control endothelial cell sprouting and angiogenesis [9,35]. Given their regulatory role in angiogenesis, these molecules have been implicated in the pathophysiology of different disorders including cancer, diabetic retinopathy, and nephropathy, as well as autoimmune diseases such as multiple sclerosis, rheumatoid arthritis, and systemic lupus erythematosus [9,11,12,13,14,15,16,17]. As far as SSc is concerned, our previous in vitro findings demonstrated a significant contribution of the neurovascular guidance molecules NRP1, Sema3E, and Slit2 in the dysregulation of the angiogenic process [9,18,20,21,22]. Moreover, NRP1 has been recently suggested as a potential biomarker to identify SSc patients who are at risk of developing pulmonary arterial hypertension [19]. Our current data strikingly fits into this scenario, adding both sNRP1 and Sema3E to the considerable list of potential circulating biomarkers that are useful to monitor the degree and, possibly, the progression of SSc peripheral microvasculopathy, with an increase in the serum Sema3E better reflecting early microvascular involvement, while a decrease in the serum sNRP1 mirroring more severe microvascular changes which can result in the development of complications such as ischemic DUs. Interestingly, besides SSc, which is known to be characterized by peripheral microvascular dysfunction culminating in capillary loss not compensated by sufficient angiogenesis, alterations in NRP1 and Sema3E protein levels have also been reported in several types of cancer, a pathologic condition that is instead characterized by excessive angiogenesis [36,37,38,39,40,41,42,43]. In the circulation, increased sNRP1 levels were associated with poor prognosis in patients with melanoma and early breast cancer [37,38], while to our knowledge, no data about serum Sema3E in tumors have been reported so far. Thus, the evidence that serum levels of Sema3E may reflect the severity of SSc-related microvascular disease may pave the way for future studies that are aimed at assessing this neuroendothelial molecule in the circulation of patients with different pathologies that are characterized by either an excess or lack of angiogenesis.

Our findings that both sNRP1 and Sema3E circulating levels show a moderate diagnostic accuracy for SSc, as revealed by ROC curve analysis and AUC estimation, also deserve attention. Indeed, if circulating sNRP1 has already been proposed as a possible diagnostic biomarker for different diseases such as cervical cancer, cervical intraepithelial neoplasia, and hepatocellular carcinoma [36,39], to our knowledge here we explored for the first time the diagnostic power of circulating Sema3E. It is worth noting that previous studies from our group reported altered levels of circulating sNRP1 and Sema3E also in subjects who were suffering from RP, with decreased sNRP1 in patients with a very early diagnosis of SSc (VEDOSS) and elevated Sema3E in subjects with primary RP [20,21]. Thus, we believe that further insights into the potential diagnostic value of these markers could arise from future longitudinal studies monitoring changes in circulating levels of sNRP1 and Sema3E in VEDOSS patients who progress into an established SSc disease. In addition, considering the cross-sectional nature of our data, we acknowledge that further prospective studies on large cohorts of SSc patients are required to ascertain whether, over time, changes in serum sNRP1 and Sema3E may correlate with the progression of NVC abnormalities and the development of certain complications such as DUs and gangrene, and whether sNRP1 and Sema3E levels at diagnosis may be predictive of a more severe peripheral vascular disease course, possibly helping in the choice of treatment. In particular, future longitudinal analyses could help to clarify the usefulness of circulating Sema3E as a biomarker, especially when considering that our data do not seem to suggest a one-way transition of its levels along with the disease development and progression. Finally, we are confident that our promising results will stimulate further research that is aimed at unveiling the possible contribution of additional members of neurovascular guidance molecule families to the pathophysiology and clinical phenotype of SSc.

## Figures and Tables

**Table 1:** Demographic, clinical, and serological characteristics of SSc patients.

Characteristics	SSc Patients (*n* = 166)
Mean ± SD age, years	58.6 ± 13.7
Sex	
Male	22 (13.2)
Female	144 (86.8)
Disease subset	
limited cutaneous SSc	111 (66.9)
diffuse cutaneous SSc	55 (33.1)
Autoantibody positivity	
Antinuclear	153 (92.1)
Anticentromere	81 (48.8)
Antitopoisomerase I	57 (34.3)
Digital ulcers	52 (31.3)
NVC pattern	
Early	53 (32.0)
Active	69 (41.5)
Late	44 (26.5)

Except where indicated otherwise, values are *n* (%) of subjects. NVC, nailfold videocapillaroscopy; SSc, systemic sclerosis.

**Table 2:** Logistic regression analysis model combining serum sNRP1, Sema3E, and Slit2 levels.

		Active/Late NVC	DUs
sNRP1	OR (95% CI)	0.65 (0.50–0.83)	0.48 (0.35–0.66)
	*p*	<0.001	<0.001
Sema3E	OR (95% CI)	0.07 (0.02–0.21)	0.09 (0.02–0.30)
	*p*	<0.001	<0.001
Slit2	OR (95% CI)	0.99 (0.97–1.01)	0.99 (0.99–1.00)
	*p*	0.335	0.479

CI, confidence interval; DUs, digital ulcers; NVC, nailfold videocapillaroscopy; OR, odds ratio.

## Data Availability

All relevant data are included within the manuscript.
